# Avian malaria co-infections confound infectivity and vector competence assays of *Plasmodium homopolare*

**DOI:** 10.1007/s00436-018-5924-5

**Published:** 2018-05-29

**Authors:** Jenny S. Carlson, Brittany Nelms, Christopher M. Barker, William K. Reisen, Ravinder N. M. Sehgal, Anthony J. Cornel

**Affiliations:** 1 Department of Entomology, University of California at Davis, Davis, CA USA; 2 Lake County Vector Control, Lakeport, CA USA; 3 Department of Pathology, Microbiology, and Immunology, University of California at Davis, Davis, CA USA; 4 Department of Biology, San Francisco State University, San Francisco, CA USA; 5 Vector Genetics Laboratory, Dept. Pathology, Microbiology and Immunology, University of California at Davis, Davis, CA USA; 6 Adjunct Appointment, School of Health Systems & Public Health, University of Pretoria, Pretoria, South Africa

**Keywords:** Avian malaria, Experimental infection, Co-infection, *Plasmodium cathemerium*, *Plasmodium homopolare*, *Culex* mosquitoes

## Abstract

Currently, there are very few studies of avian malaria that investigate relationships among the host-vector-parasite triad concomitantly. In the current study, we experimentally measured the vector competence of several *Culex* mosquitoes for a newly described avian malaria parasite, *Plasmodium homopolare*. Song sparrow (*Melospiza melodia*) blood infected with a low *P*. *homopolare* parasitemia was inoculated into a naïve domestic canary (*Serinus canaria* forma *domestica*). Within 5 to 10 days post infection (dpi), the canary unexpectedly developed a simultaneous high parasitemic infection of *Plasmodium cathemerium* (Pcat6) and a low parasitemic infection of *P*. *homopolare*, both of which were detected in blood smears. During this infection period, PCR detected Pcat6, but not *P*. *homopolare* in the canary. Between 10 and 60 dpi, Pcat6 blood stages were no longer visible and PCR no longer amplified Pcat6 parasite DNA from canary blood. However, *P*. *homopolare* blood stages remained visible, albeit still at very low parasitemias, and PCR was able to amplify *P*. *homopolare* DNA. This pattern of mixed Pcat6 and *P*. *homopolare* infection was repeated in three secondary infected canaries that were injected with blood from the first infected canary. Mosquitoes that blood-fed on the secondary infected canaries developed infections with Pcat6 as well as another *P*. *cathemerium* lineage (Pcat8); none developed PCR detectable *P*. *homopolare* infections. These observations suggest that the original *P*. *homopolare*-infected songbird also had two un-detectable *P*. *cathemerium* lineages/strains. The vector and host infectivity trials in this study demonstrated that current molecular assays may significantly underreport the extent of mixed avian malaria infections in vectors and hosts.

## Introduction

In vector-borne disease systems, identifying the relative contribution of different vector and host species is a crucial step in determining the transmission rates of pathogens in a community (McCallum et al. 2001). The task of separating minor from major vectors is relatively easily accomplished in simple avian malaria systems such as in the Hawaiian archipelago, where one *Plasmodium* and few mosquito species co-exist (Van Riper et al. 1986; LaPointe et al. 2012; Winchester and Kapan 2013). Far more complex vector-vertebrate and parasite-host-vector interactions occur in systems of multiple host and vector species (Dietz 1980). In most locations, several vectors contribute to the transmission of multiple avian pathogens, and in the context of disease dynamics, the general dimension of vector functional diversity is an important consideration. However, Power and Flecker (2008) state that functional diversity includes many factors not necessarily related just to vector taxonomic diversity. Compatibility with both the host and the vector, along with abiotic factors (such as environmental constraints and temperature), will determine the biogeographical distribution of parasites and is a product of co-evolution between parasites, hosts, and vectors (Kawecki 1998). Additionally, all parameters of the vectorial capacity of one vector species may vary considerably in time and space due to genetic polymorphisms in different populations and ecosystems (Lambrechts et al. 2009).

We attempted to investigate avian malaria dynamics in central California, where large numbers of resident and migratory songbirds and multiple *Culex*, *Culiseta*, and *Aedes* mosquito species occur. Prevalence studies conducted in 2011 and 2012 showed that China Creek Park harbored a rich diversity of avian *Plasmodium* species and lineages in birds (Walther et al. 2016) and mosquitoes (Carlson et al. 2015). However, there was some incongruence between the prevalence of *Plasmodium* species in resident birds and mosquitoes. Some parasite species identified in resident birds were not found in the local mosquitoes and vice versa. One parasite species, *Plasmodium homopolare* (belonging to the subgenus *Novyella*), newly described by Walther et al. (2014), was the most common parasite in resident birds but was rarely found in mosquitoes (Walther et al. 2014; Carlson et al. 2015). This anomaly led us to hypothesize that some ornithophilic *Culex* mosquito species were incompatible vectors of some *Plasmodium* parasite species. To test this hypothesis, we attempted to infect *Culex* mosquito species with *P*. *homopolare*. Because we were unable to culture *P*. *homopolare*, we collected this parasite from a wild bird that was identified as positive for *P*. *homopolare* by microscopy and polymerase chain reaction (PCR). In a controlled laboratory setting, this blood extract was injected into a domestic canary (*Serinus canaria* forma *domestica*). Blood from this canary was subsequently injected into three other canaries, which were in turn used to infect mosquitoes to determine vector competence.

In nature, multiple instances and opportunities occur within the vertebrate and invertebrate hosts for complex parasite-parasite interactions that ultimately impact the heterogeneity, persistence, and transmission dynamics of *Plasmodium* in a community*.* Here, we describe the results from the vector-competence studies and discuss how these data align with the prevalence data collected in the field at China Creek Park.

## Materials and methods

### Infected wild bird blood collection

Infected blood from one song sparrow (*Melospiza melodia*) was obtained from China Creek Park, Central California, following methods described by Walther et al. (2016). Following protocols designed by Carlson et al. (2016), 100 μl of song sparrow blood was extracted by jugular venipuncture with a syringe preloaded with 0.014 cc of citrate phosphate dextrose adenine (CPDA) solution to prevent clotting. Half of the blood drawn into the syringe was discharged into a tube containing lysis buffer (10 mM Tris-HCL, pH 8.0, 100 mM EDTA, 2% SDS) and held at room temperature for later DNA extraction and molecular testing. The syringe holding the remainder of the blood was placed in a plastic bag and held on top of wet ice for transport to the laboratory (for 174 miles). Two thin blood smear slides air-dried and fixed in absolute methanol in the field were also made from small aliquots of the song sparrow blood. The slides were stained, the same day the smear was made, with Giemsa as described by Valkiūnas et al. (2008). The intensity of parasitemia, estimated by counting the number of parasites per 10,000 erythrocytes, was determined to be 0.0002%. Parasites were identified both by microscopy using morphological keys described by Valkiūnas (2005) and by sequencing parasite DNA after amplification via PCR. Parasite DNA was extracted from the whole blood sample following the DNeasy blood and tissue kit protocols (Qiagen, Valencia, CA). A nested PCR described in Waldenström et al. (2004) was carried out to amplify a 478-bp sequence of the mitochondrial cytochrome *b* gene (cyt *b*). All PCR products were viewed on 1.8% agarose gels stained with ethidium bromide. The positive sample was cleaned and sent for sequencing to Elim Biopharmaceuticals Inc. (Hayward, CA). The sequences were edited using Sequencher 5.1 (Gene Codes, Ann Arbor, MI) and were then identified using a NCBI nucleotide BLAST^™^ search.

### Inoculation of donor blood into canaries

**Fig. 1:**
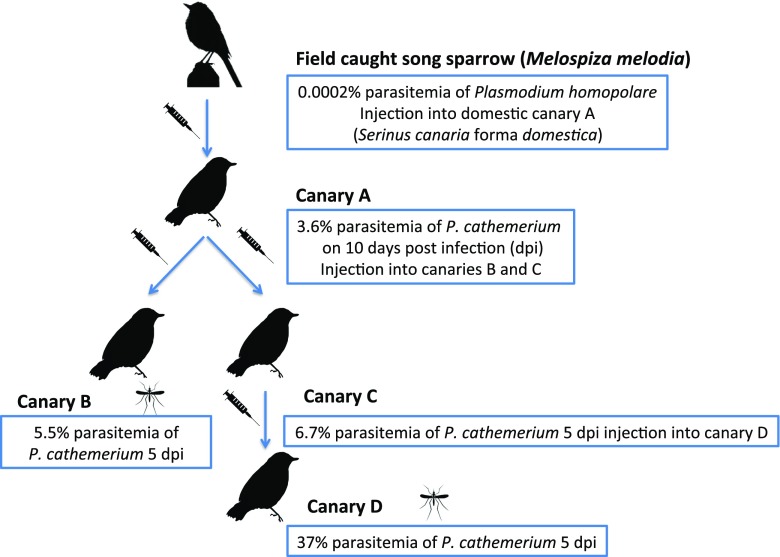
Schematic of *Plasmodium homopolare* passage from a field-caught song sparrow (*Melospiza melodia)* to naïve domestic canaries (*Serinus canaria* forma *domestica*). A mosquito symbol indicates the canaries upon which experimental mosquitoes took an infectious blood meal (see Table 1 for more details)

Microscopic examinations and PCR screening of the peripheral blood of all four domestic canaries (approximately 2–3 years of age), obtained from a California breeder (Steve Mieser, as described in our IACUC permit 17601) and used for the infectivity studies, revealed that they were not infected with malaria parasites prior to the experimentation. We confirmed with the breeder that the canaries were not exposed to mosquitoes because they were maintained in an indoor aviary. The canaries were tested upon arrival and again 15 and 30 days after arrival via blood smear inspection and PCR analysis of collected blood samples. One of the canaries (canary A) was injected with 0.04 cc of infected song sparrow whole blood into the jugular vein. Ten days post infection (dpi), a blood smear was made with whole blood of canary A extracted from the brachial vein to check for the establishment of an infection. Infected blood from canary A was injected into canaries B and C as a second passage. One last direct canary-to-canary inoculation was conducted from canary C to canary D (a third passage)—refer to Fig. 1 for the schematic.

### Collection and rearing of field mosquitoes

Adult female mosquitoes used in the experimental infections included *Culex quinquefasciatus* (colony mosquitoes obtained from the Greater Los Angeles County Vector Control District), *Cx*. *pipiens* complex (collected at China Creek Park*,* Fresno County, 36° 43′ 27.0″ N, 119° 30′ 07.3″ W), and *Cx*. *stigmatosoma* (collected by the Lake County Vector Control District at the Steele Winery, 39° 59′ 35.999″ N, 122° 52′ 31″ W). Wild *Cx*. *tarsalis* were originally collected at the Yolo Bypass Wildlife Area (38° 33′ 53.01″ N, 121° 35′ 41.621″ W), but because they all died within a few days after imbibing blood from the canaries, colony mosquitoes for this species were obtained at the Sutter-Yuba Mosquito and Vector Control District. F1 adults were reared from egg rafts collected from the surface of ponds and also from eggs laid from gravid females, which were originally collected in gravid traps (Cummings 1992; Reiter 1987). *Cx*. *pipiens* complex member species were identified by PCR, following the protocol described by Smith and Fonseca (2004), as *Cx*. *pipiens*, *Cx*. *quinquefasciatus*, and hybrids of the two species. However, we use caution with these identifications for Kothera et al. (2013) were not able to find pure *Cx*. *pipiens* in California, only hybrids.

### Experimental infection of mosquitoes

All adult mosquitoes were maintained in an incubator at 26 °C and 70% humidity with an automatic 12 h light/dark cycle. Up to 50 mosquitoes were placed in each one-gallon bucket and were provided with four cotton balls lightly soaked in 10% sucrose that were replaced daily.

To increase the probability of a successful blood feeding, all mosquitoes were starved by removing their sugar access 24 h prior to blood feeding on the infected canaries. Mosquitoes were aspirated from their original cage to a 3.7-l bucket that contained the unrestrained infected canary. The canaries were unrestrained to reduce stress on the bird that was infected with malaria and allow for a more natural feeding. The mosquitoes were allowed to feed for 1 h on each canary starting from 20 to 2100 h to emulate the crepuscular feeding pattern. The feeding was supervised for the entire hour to ensure that no more than ~ 50 mosquitoes (half of the mosquitoes in the cage) fed on one canary at a time. We determined that up to 100 mosquitoes could safely feed on a canary at a time since each mosquito can take a maximum of 3 μl of blood per feeding (Klowden and Lea 1978). A canary weighs on average 30 g so if 100 mosquitoes take 3 μl of blood, then a maximum of 300 μl will be taken in total for each feeding, which is 1% of the body weight of a bird. However, a maximum cut-off was not necessary because it was rare that more than 15 mosquitoes at a time would feed on the same canary.

Fully blood-fed mosquitoes were transferred into smaller half-liter cartons (no more than 25 individuals per carton) and held in the incubator. Mosquitoes that did not feed were placed back into their original 3.7-l container and were used again in subsequent feeding attempts, while partially blooded mosquitoes were discarded. Fully blood-fed mosquitoes were monitored daily, and any individuals that died prior to or between scheduled time points for dissections (15, 20, and 25 dpi) were placed into 70% ethanol until processed with the experimental mosquito samples.

### Determination of infection in experimental mosquitoes

To determine the infectious status, some females of each mosquito species were dissected on 15, 20, and 25 days post infection (dpi). A period of 15 days at 26 °C was considered long enough for sporozoites to have migrated to the salivary glands based on vector competence trials of other *Plasmodium* species (Meyer and Bennet 1976; Kazlauskiené et al. 2013; Carlson et al. 2016). The number of mosquitoes dissected depended on the availability of mosquitoes of each species still surviving after 15 to 25 dpi. All mosquitoes were first anesthetized with triethylamine (Sigma-Aldrich, St. Louis, MO). Salivary glands were dissected from the thorax and the midgut from the abdomen, and slide preparations made. Thoraxes and salivary glands were placed in separate tubes containing 70% ethanol and were used for parasite DNA extraction and amplification in the same manner described by Carlson et al. (2015). Preparations of midguts to detect oocysts were made by removing the midgut and placing it into a drop of saline, followed by adding a drop of 0.5% solution of mercurochrome. Because no permanent preparations of midguts were made, each midgut was viewed microscopically within 20 min of preparation.

The ideal way to determine vector competence is to have infected vectors refeed on a naïve host. We attempted refeeding mosquitoes on two *Plasmodium-*negative canaries, but every attempt to get mosquitoes to refeed failed, despite providing an opportunity for the mosquitoes to oviposit eggs developed after their primary (first infective) blood meal. Therefore, we were forced to use an artificial capillary tube method (Aitken 1977; Cornel et al. 1993) to test for sporozoites secreted in saliva extrapolates at 15, 20, and 25 dpi. Initially, we filled each capillary with a solution containing equal parts of heat-inactivated fetal bovine serum (FBS; Sigma-Aldrich, St. Louis, MO) and 10% sucrose. However, after constant parasite-negative results, despite using mosquitoes with known parasite-positive salivary glands, we concluded that this method had to be modified for *Plasmodium* studies. We then used a modified version of the Rosenberg et al. (1990) method, which did provide PCR-positive saliva samples. In this method, the fascicle sheath of the mosquito was first removed and the exposed proboscis was then inserted into the capillary tubes filled with a mixture of one part mineral oil and one part 10% sucrose. The mosquito was allowed to expectorate for 15 min before being removed for further processing.

### Data analysis

Salivary gland infection rates, determined by PCR as described in Carlson et al. (2015), were tested for variation between mosquito species using a logistic regression analysis. The analysis modeled the proportion of positive salivary glands against the day of infection and tested whether the overall proportion of mosquitoes with sporozoites in their salivary glands differed between mosquito species after adjustment for any time trend. All analyses were performed in R version 3.2 (R Core Team 2015).

## Results

### Canary infections

Canary A was believed to be infected with only *P*. *homopolare* (lineage SOSP_CA3P; GenBank accession number KJ482708); however, recipient canary A was subsequently found to be co-infected with both *P*. *homopolare* and *P*. *cathemerium*. *P*. *cathemerium* lineage SPTO_CA_ELW_6P (Pcat6; GenBank accession number KJ620779) infection with 3.6% merozoites was first detected on 10 dpi in canary A, and gametocytes were detected on 5 dpi in the two secondary infected canaries (B and C). *P*. *cathemerium* parasitemias in canaries B and C ranged from 5.5 to 6.7%, respectively. Canary D, which was infected with blood from canary C, had the highest *P*. *cathemerium* parasitemia on 5 dpi at 37%. In all canaries, *P*. *cathemerium* parasitemias declined after 5 dpi to < 1% on 7 dpi and to < 0.2% on 9 dpi. In all four canaries, only two or fewer *P*. *homopolare* gametocytes were visualized per 1000 erythrocytes (≤ 0.002%) on days 5, 7, and 9 post infection. It was possible that some trophozoites that we called *P*. *cathemerium* may have been *P*. *homopolare* trophozoites, because it is very difficult to differentiate trophozoites of the two species morphologically (Valkiūnas 2005). All canaries survived the experimental infection, and only after 2 months did they test positive for *P*. *homopolare* by PCR. Microscopically, trophozoites of *P*. *homopolare* were still visible in the blood, indicating that a chronic infection persisted. *P*. *cathemerium* was no longer detected by PCR or seen in blood smears after 2 months post-infection. Infection with a third parasite lineage became apparent xenodiagnotically only when the experimentally infected mosquitoes were tested for parasite DNA. This third parasite had a cyt *b* sequence identical to *P*. *cathemerium* lineage HOFI_CA_ELW_8P (Pcat8; GenBank accession number KJ620781) that was previously reported by Carlson et al. (2015) as a *P*. *cathemerium-*like lineage isolated from mosquitoes and birds in China Creek Park. Pcat6 and Pcat8 lineages have a genetic distance of 0.85%.

### Mosquito infections

A total of 286 mosquitoes fully blood-fed on *Plasmodium* infected canaries B and D between 5 and 25 dpi. Of these blood-fed mosquitoes, 115 were dissected at 15, 20, and 25 dpi (Table 1), of which 44 were infected with *Plasmodium* (37%). The other 169 blood-fed mosquitoes died before or in between the time points and could not be dissected, but were preserved in 70% ethanol. The majority (153/169) of the mosquitoes that died were *Cx*. *tarsalis* that had been collected from the field*.* Of the 169 dead mosquitoes, 66 (39%) thoraces were positive when tested by PCR. Table 1 shows the differences between the feeding patterns on canaries B and D. *Cx*. *stigmatosoma* did not readily take a blood meal from canary B, despite having equal opportunity to feed on this canary as other mosquito species.

**Table 1:** Table 1 reports mosquito infections tested on 15, 20, and 25 dpi resulting from blood meals obtained from canary B and canary D, respectively. For each of the three time points, the infections are reported for each of the two *Plasmodium cathemerium* lineages Pcat6 (SPTO_CA_ELW_6P, GenBank accession number KJ620779) and Pcat8 (HOFI_CA_ELW_8P, GenBank accession number KJ620781). For each of the five mosquito species tested, the total number of positive thoraxes (*T*) and salivary glands (*S*) are reported, followed by the total sample size (*N*). The symbol “-” characterizes samples for which a PCR was not carried out because there were not enough mosquito specimens to test at that time point

Canary B		Mosquito species														
	*Plasmodium cathemerium* lineage	*Cx*. *pipiens*			*Cx*. *quinquefasciatus*			*Cx*. *quinquefasciatus-pipiens* hybrids			*Cx*. *stigmatosoma*			*Cx*. *tarsalis*		
DPI		*T* (+)	*S* (+)	*N*	*T* (+)	*S* (+)	*N*	*T* (+)	*S* (+)	*N*	*T* (+)	*S* (+)	*N*	*T* (+)	*S* (+)	*N*
15	SPTO_CA_ELW_6P	-	-	0	-	-	0	-	-	0	-	-	0	3	1	3
	HOFI_CA_ELW_8P	-	-		-	-		-	-		-	-		0	0	
20	SPTO_CA_ELW_6P	0	0	2	2	0	10	1	1	7	-	-	0	1	1	1
	HOFI_CA_ELW_8P	0	0		0	0		0	0		-	-		0	0	
25	SPTO_CA_ELW_6P	0	0	2	3	0	10	0	0	4	-	-	0	1	1	2
	HOFI_CA_ELW_8P	0	0		0	1		0	0		-	-		1	1	
	Total	0	0	4	5	1	20	1	1	11	-	-	0	6	4	6
Canary D		Mosquito species														
	*Plasmodium cathemerium* lineage	*Cx*. *pipiens*			*Cx*. *quinquefasciatus*			*Cx*. *quinquefasciatus-pipiens* hybrids			*Cx*. *stigmatosoma*			*Cx*. *tarsalis*		
DPI		*T* (+)	*S* (+)	*N*	*T* (+)	*S* (+)	*N*	*T* (+)	*S* (+)	*N*	*T* (+)	*S* (+)	*N*	*T* (+)	*S* (+)	*N*
15	SPTO_CA_ELW_6P	0	0	1	0	0	10	0	0	4	2	1	12	7	6	10
	HOFI_CA_ELW_8P	0	0		0	0		0	0		8	4		0	0	
20	SPTO_CA_ELW_6P	-	-	0	0	0	7	0	0	2	0	3	7	6	6	8
	HOFI_CA_ELW_8P	-	-		0	0		0	0		5	0		1	1	
25	SPTO_CA_ELW_6P	-	-	0	0	0	6	0	0	2	0	0	3	1	1	2
	HOFI_CA_ELW_8P	-	-		0	0		0	0		1	0		1	1	
	Total	0	0	1	0	0	23	0	0	8	16	8	22	16	15	20

**Fig. 2:**
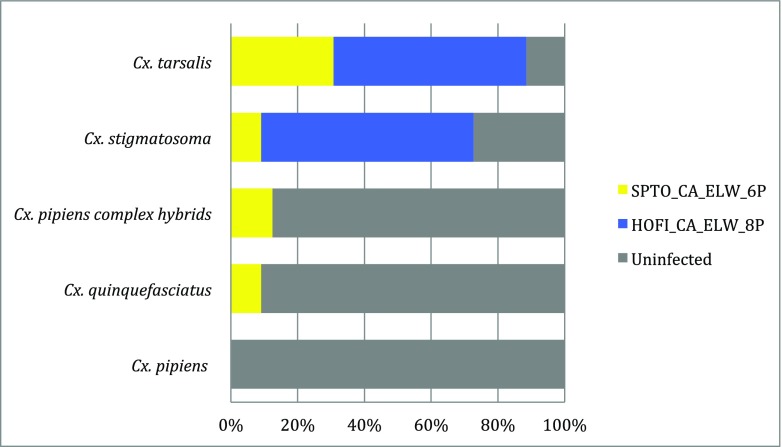
Prevalence (reported as total % of all mosquitoes tested at 15, 20, and 25 dpi) of *Plasmodium cathemerium* lineages Pcat6 (SPTO_CA_ELW_6P, GenBank accession number KJ620779) and Pcat8 (HOFI_CA_ELW_8P, GenBank accession number KJ620781) in the five mosquito species tested. No mosquitoes were infected with *P*. *homopolare*. No infections were observed in *Cx*. *pipiens*. *Cx*. *tarsalis* presented the highest prevalence of all five species. *P*. *cathemerium* lineage Pcat8 was only detected in *Cx*. *stigmatosoma* and *Cx*. *tarsalis*

**Fig. 3:**
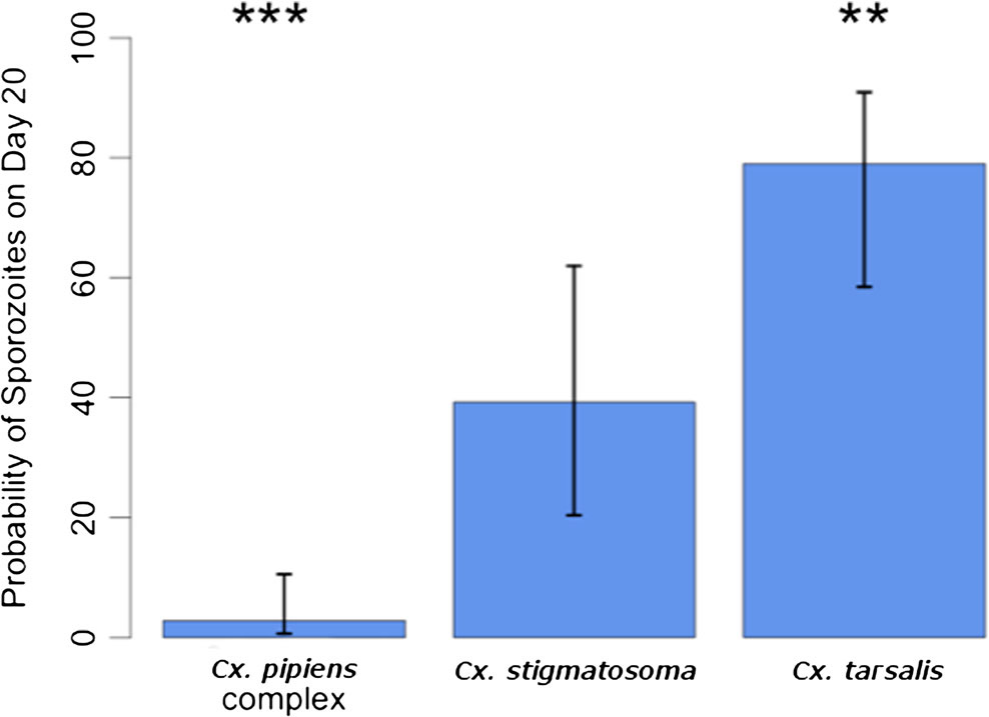
The probability of sporozoite infection by *Plasmodium* parasites (based on prevalence of salivary gland infections using logistic regression with adjustment for the trend over time post-infection for *Culex pipiens* complex, *Cx*. *stigmatosoma*, and *Cx*. *tarsalis*. Bars represent the 95% confidence intervals surrounding the mean probability of infection. The mean probability of infection at 20 dpi was 39.2% (95% CI 20.4–61.9%) for *Cx*. *stigmatosoma.* The probability of infection was significantly lower for *Culex pipiens* complex at 2.7% (*P* = 0.0004; 95% CI 0.7–10.5%) and significantly higher for *Cx*. *tarsalis* at 79.0% (*P* = 0.0063; 95% CI 58.5–91.0%) compared to that for *Cx*. *stigmatosoma*; **P < 0.01, ***P < 0.001

*Plasmodium homopolare* was not detected in any of the blood-fed mosquitoes. Fig. 2 shows the percentage of uninfected vs infected females for each species and the percentage infection with the two *P*. *cathemerium* lineages. *P*. *cathemerium* lineage Pcat6 was detected in salivary glands of all species except *Cx*. *pipiens*, whereas lineage Pcat8 was detected in only *Cx*. *stigmatosoma* and *Cx*. *tarsalis.* The probability of sporozoite infection by *P*. *cathemerium* parasites (based on salivary gland infections by PCR) differed significantly among mosquito species based on the logistic regression with adjustment for the trend over time post-infection (Fig. 3). For *Cx*. *stigmatosoma*, the mean probability of Pcat6 and Pcat8 salivary gland infection at 20 dpi was 39.2% (95% CI 20.4–61.9%). The probability of infection was significantly lower for *Cx*. *pipiens* at 2.7% (*P* = 0.0004; 95% CI 0.7–10.5%) and highest for *Cx*. *tarsalis* at 79.0% (*P* = 0.0063; 95% CI 58.5–91.0%) at 20 dpi.

Detection of sporozoites was attempted by collecting expectorate samples by the capillary tube method. Only three saliva samples collected by the modified Rosenberg et al. (1990) method described above tested positive by PCR. Two *Cx*. *stigmatosoma* and one *Cx*. *tarsalis* were positive for lineage Pcat8.

Oocysts were counted in the midguts from each mosquito at each of the three post-infection time points. Total number of oocysts ranged from 1 to 23 in *Cx*. *stigmatosoma* (*n* = 16), from 1 to 15 in *Cx*. *tarsalis* (*n* = 22), and from 1 to 28 (*n* = 6) in *Cx*. *quinquefasciatus*. The single *Cx*. *pipiens-quinquefasciatus* hybrid mosquito that tested positive had three oocysts. Because it is impossible to morphologically distinguish among *Plasmodium* species during the oocyst and sporozoite stages (Valkiūnas 2005), we were not able to determine whether these oocysts were from one or more species. One potential solution for this in future studies could be to use laser capture microdissections of midgut epithelia of mosquitoes, similar to the methods described by Lutz et al. (2016).

## Discussion

### Experimental infection

A question often asked in disease ecology is as follows: what determines pathogen infection dynamics in time and space to explain trends in maintenance and spread? It is generally agreed that the main determinants of the structuring of parasite and host associations and heterogeneity of infection in a host population are host exposure and innate and adaptive immune responses to the pathogens (Poulin 2011). Host exposure is influenced by the capacity of the local vectors to transmit the pathogens, otherwise known as vectorial capacity (Garrett-Jones and Shidrawi 1969). In studies conducted at China Creek Park (Carlson et al. 2015; Walther et al. 2016), incongruence between host and vector *Plasmodium* infection prevalences led to proposing that specific parasite-vector interactions (incompatibilities and compatibilities) were likely occurring, which could be tested by vector experimental infection assays. Experimental vector infection studies undertaken in this study revealed that mixed *Plasmodium* infections, which are common in hosts (Manwell and Herman 1935; Dimitrov et al. 2015; Palinauskas et al. 2011), raise new interpretative challenges and parasite species interactions in vectors, which can determine relative parasite abundance in time and space.

One of the obvious results in this study was that none of the mosquitoes presented with *P*. *homopolare* became infected, despite imbibing gametocytes of this species, admittedly at low levels of < 0.002%. Non-exclusive hypotheses may be suggested as following: (1) this level of gametocytemia was too low for effective syngamy within the mosquito midguts precluding the infection of any species of mosquito; (2) co-infection with *P*. *cathemerium* prevented *P*. *homopolare* from developing an infection in the mosquito; (3) the mosquito species in this study are not the natural vector of *P*. *homopolare*; and/or (4) the PCR assay was not sensitive enough to detect *P*. *homopolare* when it was simultaneously present with the two *P*. *cathemerium* lineages.

1. *P*. *homopolare* was described in 2014 (Walther et al. 2014), and little is known about the life cycle and pathology of this species. Considering the extremely high prevalence but low parasitemia in the birds captured at China Creek (< 0.25% parasitemia, Walther et al. 2016) and in the canaries in this study (< 0.002%), it is possible that *P*. *homopolare* progresses to a long-lasting chronic infection in hosts and there is only a short window of time during which the gametocytemia is high enough to infect mosquitoes. In this study, we were unable to determine the minimum gametocytemia level required for *P*. *homopolare* to infect mosquitoes, and we may have missed the infectious window.
2. Competition with congeneric parasites limits *P*. *homopolare* infections in mosquitoes. There are opportunities for *Plasmodium* species to interact in mosquitoes, especially when vector species overlap (Paul et al. 2002) and mixed infections in infective birds are quite common (Valkiūnas et al. 2003; Biedrzycka et al. 2015). In in vitro studies, Valkiūnas et al. (2013, 2014) noted several reproductive outcomes in blood containing gametes from several avian *Haemoproteus* parasite species. Outcomes included complete blockage of development of ookinetes of some species, to increase in reproductive success (increase in ookinete production) of other species and, on occasion, production of hybrid ookinetes. Based on our infective studies, it is possible that *P*. *cathemerium* blocked syngamy and development of *P*. *homopolare* ookinetes in the species of mosquitoes that were infected. Much knowledge is lacking concerning in vivo interactions of *Plasmodium* gametes and ookinete development in mixed infections in mosquitoes, studies of which will be enhanced, when parasite species-specific nuclear markers are available which can detect hybrids (Valkiūnas et al. 2014).

Moreover, when discussing parasite-parasite competition, it is also important to consider host specificity in the context of parasites and the relationship between host breadth and host-use proficiency. *P*. *homopolare* was recently described by Walther et al. (2014) as a new species and *P*. *cathemerium* is a generalist parasite, having been detected in 9 families and 26 species, with a worldwide distribution (Valkiūnas 2005). *P*. *homopolare* infected 84 birds of 399 birds collected at China Creek Park, representing 9 host species from 5 families. A total of 31% of birds collected at China Creek Park were infected with *Plasmodium*, and 68% were infected with *P*. *homopolare*, which is the same as saying that 1 in 5 birds at China Creek Park were infected with *P*. *homopolare* (Walther et al. 2014)*. P*. *cathemerium* was found in 6 bird species and 3 families at China Creek Park. This means that both parasites are considered generalists with the ability to infect multiple distantly related host taxa and can switch between resident and migrant bird species (Waldenström et al. 2002). Little is known about the transmission cycle of *P*. *homopolare*, especially in the sense of its course of infection of the acute phase vs the chronic phase. Thus, we had no a priori knowledge on how this parasite would infect our laboratory canaries; in other words, we do not know if canaries can be naturally infected with this parasite. However, it has been reported in the human malaria field that *P*. *vivax* is suppressed during an acute infection in the presence of *P*. *falciparum*, but re-emerges as a chronic infection once *P*. *falciparum* subsided (Boyd and Kitchen 1937; Maitland et al. 1996; Bruce et al. 2000). This could be explained by immune-mediated apparent competition in a host where there are modifications to host susceptibility when a host’s immune response to one parasite affects its ability to control a second species.

3: The vector responsible for maintenance of *P*. *homopolare* in nature was not among the species used in our study. At China Creek Park, Carlson et al. (2015) reported that only 3 out of the 76 *Plasmodium*-positive field-collected mosquito thoraxes were infected with *P*. *homopolare*: one *Cx*. *tarsalis* (which was the only individual with positive salivary glands), one *Cx*. *restuans*, and one *Culiseta particeps*. However, at the same site, Walther et al. (2014) reported that 68% of infected birds were infected with *P*. *homopolare* by PCR, but not all blood smears were checked for gametocytes*.*

Combes (1991) described the two ecological drivers of heterogeneous distribution of parasites in a host population as the α and β filters. The encounter filter α refers to the ecological and/or behavioral obstacles that result in the exclusion of a parasite in a host species. The compatibility filter β refers to the successful metabolic and/or immunological response in an individual host to an invading pathogen that results in the exclusion of species that do not permit coexistence with the invading pathogen. Very few studies have tested the α and β filters directly in avian malaria vectors. According to several prior studies (Gager et al. 2008; Njabo et al. 2011; Medeiros et al. 2013; Valkiūnas et al. 2015), vectors do not play a role in driving avian *Plasmodium* parasite ranges and that ranges are determined by host compatibilities. This may not be universally true, and the range of *P*. *homopolare* may be driven by the presence of compatible vectors such as *Cx*. *restuans* in the northern hemisphere. Perhaps, there is specific vector compatibility of *P*. *homopolare* for *Cx*. *restuans*. *Cx*. *restuans* is widespread in the USA but also has a patchy distribution (Darsie and Ward 2005) with preferences for marshy areas. Several other studies provide evidence for a β filter by demonstrating genotype-by-genotype interactions between pathogens and their vectors, which then may mean that the structuring of pathogens within populations may be, in part, a result of adaptation of pathogens to local vector genotypes (Ferguson and Read 2002; Schmid-Hempel and Ebert 2003; Lambrechts et al. 2005; Joy et al. 2008; Lambrechts et al. 2009). For example, Lambrechts et al. (2009) provide experimental evidence for the potential role of vector-driven genetic structuring of dengue viruses. Clearly, for avian malaria, more studies are needed to discern the role of vectors as potential filters.

4: The apparent absence of *P*. *homopolare* in laboratory-infected mosquitoes might have resulted from deficiencies in current diagnostic tools (Bernotienė et al. 2016; Clark et al. 2016). Only after several host passages and vector infectivity studies did we discover that the original donor song sparrow was infected with three *Plasmodium* species and lineages. The determination of the infection status in the host and the vector can be done by microscopy, ELISA, and PCR-based methodologies, although each method presents challenges. There is limited worldwide expertise available to morphologically identify the erythrocytic stages of parasites, and this is particularly difficult when infections are at the early trophozoite stages and at low parasitemias. In mosquito slide preparations, it is impossible to morphologically distinguish between parasite species. ELISA and PCR-based methodologies can detect conspecific infections provided species-specific circumsporozoite (CS) proteins and primers are available (Coleman et al. 2002; Marchand et al. 2011). Species-specific CS protein and primers are not available for most avian malaria parasites, and we propose that co-infections of avian *Plasmodium* are therefore underrepresented in host and vector prevalence studies. Additionally, the PCR primer set used in this study, which was not species-specific, has been shown to preferentially amplify one parasite species or lineage over another in the presence of co-infections (Zehtindjiev et al. 2012). These vector infectivity studies confirmed that mosquitoes can support co-infections as the thorax and the salivary glands from individual mosquitoes were infected with different lineages of *P*. *cathemerium* simultaneously.

Unfortunately, we were unsuccessful in coaxing the infected mosquitoes to take a second blood meal on naïve canaries to test whether they were capable of transmitting multiple *Plasmodium* to a recipient canary. The artificial capillary method that was used to detect the expectoration of sporozoites showed some promise. However, this method likely needs considerable refinement and testing before it can be used as a reliable surrogate method for testing in vivo transmission of avian sporozoites. In addition, suitable PCR methods must be available to determine which *Plasmodium* species sporozoites have been expectorated. Future avian vector competence studies, especially when performed on non-cultured *Plasmodium* species, should use a combination of transmission assays and multiple *Plasmodium* species-specific primers on various mosquito parts and extracts especially to identify potentials of co-infections/interactions and transmissibility.

### Consequences of mixed infections and vector compatibility

Boëte and Paul (2006) stated that current species dynamics of parasites could become disturbed by control measures and may lead to epidemiological changes, but the predictability of the changes would be dependent on the level of equilibrium within the system. Because there are multiple levels in which parasites can interact with one another through competition, such as resource, interference, and immune-mediated competition, it is not clear at which point these parasites reach a state of equilibrium, if ever at all (Snounou and White 2004).

As mentioned above, the general consensus is that hosts rather than the vectors drive avian *Plasmodium* parasite geographical ranges (Medeiros et al. 2013). However, in our field system, it is not clear how *P*. *homopolare* was able to be so abundant in the resident avian population. The results from our vector competence assay indicated that there needs to be more research conducted on how competition among parasites may be driving how mosquitoes transmit the parasites. There is support for this notion in the context of mixed infections. Paul et al. (2002) proposed that interspecific competition during transmission in the vector may have contributed to a restriction of *P*. *gallinaceum* around the world. Presence of *P*. *juxtanucleare* may reduce the R_0_ of *P*. *gallinaceum* and could reduce the invasion or establishment of *P*. *gallinaceum.* Further supporting evidence can be found in examples of competition for red blood cells affecting parasitemias (McQueen and Mckenzie 2006) and gametocyte production (Bousema et al. 2008) which will alter the potential for establishing infections in mosquitoes. Because of mixed infections complicating outcomes of the vector competence trials in this study, we were unable to make tacit conclusions about the susceptibilities of mosquitoes tested for *P*. *homopolare*.

If vector compatibility differences do occur, heterogeneity in parasite ranges and temporal occurrence can be expected. Different prevalences of *P*. *vivax* phenotypes exist between Gulf of Mexico and Pacific coastlines because of differences in susceptibilities and geographic distributions between *Anopheles albimanus* (favoring phenotype VK210) and *An*. *pseudopunctipennis* (favoring phenotype VK247) (Rodriguez et al. 2000). Temporal fluctuations of these two *P*. *vivax* phenotypes were also noted in Thailand, and one of the explanations offered was related to seasonal abundance of susceptible vectors (Suwanabun et al. 1994). *P*. *homopolare* is likely quite widespread in the USA, based on 100% sequence matches in GenBank from various bird species (Walther et al. 2014), but on more local scales has a patchy distribution. In California, no *P*. *homopolare* parasites were seen in blood smears or detected by PCR in 200 birds in 2014 and 2015 at the Stone Lakes NWR, Elk Grove, CA (Carlson et al. unpublished data). Both China Creek and Stone Lakes share similar riverine habitat, bird species, and mosquito species except for *Cx*. *restuans*, which were not found in the latter site.

Vector competence for avian malaria parasites in the *Cx*. *pipiens* complex in California should be further studied. It is curious that along with this study, reports by Carlson et al. (2015) and by Carlson et al. (2016) identify *Cx*. *pipiens* complex as a low importance vector. Because Kothera et al. (2013) propose that *Cx*. *pipiens* are mostly hybrids of *Cx*. *pipiens* and *Cx*. *quinquefasciatus* in California, it is plausible that this genetic makeup allows them to be less permissible to avian malaria parasites in comparison to most reports on these species elsewhere in the world (Valkiūnas 2005).
